# Collaborative effects of bystander-initiated cardiopulmonary resuscitation and prehospital advanced cardiac life support by physicians on survival of out-of-hospital cardiac arrest: a nationwide population-based observational study

**DOI:** 10.1186/cc9319

**Published:** 2010-11-04

**Authors:** Hideo Yasunaga, Hiromasa Horiguchi, Seizan Tanabe, Manabu Akahane, Toshio Ogawa, Soichi Koike, Tomoaki Imamura

**Affiliations:** 1 Department of Health Management and Policy, Graduate School of Medicine, The University of Tokyo, 7-3-1 Hongo, Bunkyo-ku, Tokyo 113-8655, Japan; 2 Foundation for Ambulance Service Development, Emergency Life-Saving Technique Academy of Tokyo, 4-5 Minami-osawa, Hachioji, Tokyo 192-0364, Japan; 3 Department of Public Health, Health Management and Policy, Nara Medical University School of Medicine, 840 Shijocho, Kashihara, Nara 634-8521, Japan; 4 Department of Planning, Information and Management, The University of Tokyo Hospital, 7-3-1 Hongo, Bunkyo-ku, Tokyo 113-8655, Japan

## Abstract

**Introduction:**

There are inconsistent data about the effectiveness of prehospital physician-staffed advanced cardiac life support (ACLS) on the outcomes of out-of-hospital cardiac arrest (OHCA). Furthermore, the relative importance of bystander-initiated cardiopulmonary resuscitation (BCPR) and ACLS and the effectiveness of their combination have not been clearly demonstrated.

**Methods:**

Using a prospective, nationwide, population-based registry of all OHCA patients in Japan, we enrolled 95,072 patients whose arrests were witnessed by bystanders and 23,127 patients witnessed by emergency medical service providers between 2005 and 2007. We divided the bystander-witnessed arrest patients into Group A (ACLS by emergency life-saving technicians without BCPR), Group B (ACLS by emergency life-saving technicians with BCPR), Group C (ACLS by physicians without BCPR) and Group D (ACLS by physicians with BCPR). The outcome data included 1-month survival and neurological outcomes determined by the cerebral performance category.

**Results:**

Among the 95,072 bystander-witnessed arrest patients, 7,722 (8.1%) were alive at 1 month, including 2,754 (2.9%) with good performance and 3,171 (3.3%) with vegetative status or worse. BCPR occurred in 42% of bystander-witnessed arrests. In comparison with Group A, the rates of good-performance survival were significantly higher in Group B (odds ratio (OR), 2.23; 95% confidence interval, 2.05 to 2.42; *P *< 0.01) and Group D (OR, 2.80; 95% confidence interval, 2.28 to 3.43; *P *< 0.01), while no significant difference was seen for Group C (OR, 1.18; 95% confidence interval, 0.86 to 1.61; *P *= 0.32). The occurrence of vegetative status or worse at 1 month was highest in Group C (OR, 1.92; 95% confidence interval, 1.55 to 2.37; *P *< 0.01).

**Conclusions:**

In this registry-based study, BCPR significantly improved the survival of OHCA with good cerebral outcome. The groups with BCPR and ACLS by physicians had the best outcomes. However, receiving ACLS by physicians without preceding BCPR significantly increased the number of patients with neurologically unfavorable outcomes.

## Introduction

Successful rescue of patients with cardiac arrest outside the hospital setting is a long-term public health issue in most jurisdictions in the majority of countries [1,2]. Reported out-of-hospital cardiac arrest (OHCA) survival rates vary widely [2-8], and this variation can be attributed, in part, to differences among countries in the chain of survival, as described by the American Heart Association [9]. Ideally, communities and emergency medical service (EMS) providers should optimize the following four links: rapid access through an emergency telephone system; early bystander-initiated cardiopulmonary resuscitation (BCPR) [10]; early defibrillation with an automated external defibrillator [11]; and advanced cardiac life support (ACLS) [12,13].

Numerous studies have elucidated the independent effects of BCPR coupled with use of an automated external defibrillator [11]. On the other hand, the extent to which prehospital ACLS can improve patient survival remains somewhat controversial [7,10,12,13], even more than 40 years after Pantridge and Geddes introduced the concept of providing ACLS to OHCA patients through mobile intensive care vehicles [1]. The issue of who provides the prehospital ACLS is considered one of the factors affecting outcomes after OHCA. Several studies have assessed the effects of physician-manned ACLS [12,13], but the studies were conducted in limited geographic areas. Furthermore, the relative importance of BCPR and ACLS and the effectiveness of their combination on the survival rate of patients and their subsequent well-being have not been clearly demonstrated.

The aims of the present study were to analyze the collaborative effects of BCPR and prehospital ACLS with or without physicians on the health outcomes of OHCA patients, in terms of their overall survival and cerebral performance at 1 month.

## Materials and methods

### Emergency medical service in Japan

In Japan, the fire defense headquarters of local governments - which comprised 807 fire stations with dispatch centers as of 2007 - provide the standardized prehospital EMS. The Fire and Disaster Management Agency of Japan (FDMA) supervises the EMS system throughout the nation. Generally, an ambulance crew is organized with three EMS staff members in a local center, including at least one emergency life-saving technician (ELST) who has undergone extensive training for providing prehospital EMS [11,14]. ELSTs perform cardiopulmonary resuscitation (CPR) according to the Japanese CPR guidelines, which are based on the guidelines of the American Heart Association and the International Liaison Committee on Resuscitation [15,16]. ELSTs provide prehospital EMS procedures that are limited to use of a semiautomated external defibrillator, insertion of an adjunct airway (esophageal obturator airway or laryngeal mask airway), cannulation of a peripheral intravenous line and infusion of lactate Ringer solution and epinephrine. Only specially trained ELSTs are permitted to insert tracheal tubes [14].

The Japanese EMS system is one-tiered. Several regions have their own physician-staffed EMS systems [14,17]. On receipt of an emergency call to a dispatch center in such regions, the EMS personnel request mobilization of a physician-staffed ambulance from an emergency medical center if the patient is suspected of OHCA [18]. Physician-manned ambulances, although increasing in number, are not yet widespread in Japan. As of April 2009, there were approximately 4,000 hospitals with an emergency room and 218 emergency and critical care centers in all 47 prefectures in Japan. Eighty-six of the 218 centers organized physician-staffed prehospital ACLS systems [19]. A previous questionnaire survey showed that 27 out of 48 physician-staffed ambulance systems worked 24 hours a day while the other 21 systems only worked during the daytime in 2006 [18]. In physician-manned ambulances, prehospital physicians can carry out any emergency treatment according to their diagnoses and judgments, and can select many treatment options including use of a semiautomated external defibrillator, tracheal tube insertion, central venous catheterization, and infusion of catecholamines, lidocaine, atropine, anesthetic drugs and thrombolytic agents. In-hospital treatment after return of spontaneous circulation varies widely between hospitals. In particular, hospitals with physician ACLS typically provide more optimal post-return of spontaneous circulation treatments, including therapeutic hypothermia and percutaneous coronary intervention.

### Data source

In January 2005, the FDMA launched a prospective, nationwide, population-based, observational study involving all OHCA patients in Japan [11]. The EMS staff in each center recorded the data of OHCA patients using an Utstein-style form [18] in cooperation with the physicians in charge of the patients. The anonymous data were sent electronically to the FDMA database server. The database included the following data: sex; age; cause of arrest (cardiac or noncardiac origin); bystander's witness status; presence of BCPR; times of collapse, emergency call, vehicle arrival at the scene, initiation of CPR and first shock; initial rhythms, including ventricular fibrillation (VF), pulseless electrical activity and asystole; information on the EMS crew (physician-staffed or not); 1-month survival; and neurological outcome 1 month after cardiac arrest, defined as the Glasgow-Pittsburgh cerebral performance category (CPC) [9,20]. The physician in charge made a diagnosis of the cause of arrest in collaboration with the EMS staff.

The FDMA offered all of the anonymous data to our research group. The present study was approved by the Institutional Review Board of Nara Medical University.

### Subjects

In the present study, we enrolled all OHCA patients who were witnessed by laypeople or EMS providers, who received or did not receive BCPR, who underwent prehospital ACLS by ELSTs or physicians and who were transported to medical facilities from 1 January 2005 to 31 December 2007. While a witnessed arrest status is one of the strong prognostic factors for survival of OHCA, an unwitnessed arrest is associated with little possibility of survival [9]. In the present study, patients were excluded from the analysis if their cardiac arrest was not witnessed or if the witness status was not documented.

We divided the bystander-witnessed OHCA patients into the following four groups: patients who did not undergo BCPR and were treated with prehospital ACLS by ELSTs (Group A: ACLS by ELSTs without BCPR); patients who underwent BCPR and were treated with prehospital ACLS by ELSTs (Group B: ACLS by ELSTs with BCPR); patients who did not undergo BCPR and were treated with prehospital ACLS by physicians (Group C: ACLS by physicians without BCPR); and patients who underwent BCPR and were treated with prehospital ACLS by physicians (Group D: ACLS by physicians with BCPR).

For the EMS provider-witnessed OHCA patients, we divided the population into two groups treated with prehospital ACLS by ELSTs or by physicians.

### Endpoints

Outcome data included 1-month survival and neurological status 1 month after the event, defined by the Glasgow-Pittsburgh CPC: good performance, CPC1; moderate disability, CPC2; severe cerebral disability, CPC3; vegetative state, CPC4; or brain death, CPC5. In the database, a *de facto *brain-death case was considered still alive if the patient had not been diagnosed with the standard diagnostic criteria for brain death but was coded as CPC5.

### Analyses

We performed univariate comparisons of the patient characteristics and outcome variables using chi-square tests and analysis of variance as appropriate. Logistic regression analyses were performed to model the concurrent effects of BCPR, ACLS and other factors on the outcomes. The threshold for significance was *P *< 0.05. All statistical analyses were conducted using PASW Statistics version 18.0 (SPSS Inc., Chicago, IL, USA).

## Results

During the study period, we identified 95,072 bystander-witnessed OHCA patients (29,215 in 2005, 31,849 in 2006 and 34,008 in 2007) and 23,127 EMS provider-witnessed OHCA patients (7,554 in 2005, 7,717 in 2006 and 7,856 in 2007).

Table 1 presents demographic data of the bystander-witnessed OHCA patients. Overall, 39,993 patients (42.1%) received BCPR and 3,513 patients (3.7%) received ACLS by physicians. The median times from collapse to CPR were 2 minutes in Group B and Group D with BCPR, and 10 minutes in Group A and Group C without BCPR.

**Table 1:** Baseline characteristics of patients whose arrests were witnessed by bystanders

	All (*n *= 95,072)		Group A (*n *= 53,482)		Group B (*n *= 38,077)		Group C (*n *= 1,597)		Group D (*n *= 1,916)	
					
	*n*	%	*n*	%	*n*	%	*n*	%	*n*	%
Sex (males)	58,745	61.8	34,297	64.1	22,210	58.3	1,058	66.2	1,180	61.6
Age (years)	71.6 ± 17.8 (75)		70.9 ± 17.4 (75)		72.9 ± 18.1 (77)		65.9 ± 20.1 (71)		69.2 ± 18.8 (73)	
Causes of arrest										
Cardiac origin	52,830	55.6	28,825	53.9	21,967	57.7	835	52.3	1,203	62.8
Noncardiac origin	42,242	44.4	24,657	46.1	16,110	42.3	762	47.7	713	37.2
Initial rhythm										
Ventricular fibrillation	13,705	14.4	6,744	12.6	6,292	16.5	245	15.3	424	22.1
Pulseless electrical activity	31,141	32.8	18,580	34.7	11,361	29.8	573	35.9	627	32.7
Asystole	47,328	49.8	27,129	50.7	18,724	49.2	712	44.6	763	39.8
Others	2,898	3.0	1,029	1.9	1,700	4.5	67	4.2	102	5.3
Collapse to initiation of CPR (minutes)	8.9 ± 10.0 (7.0)		12.5 ± 10.2 (10.0)		4.1 ± 7.2 (2.0)		13.0 ± 11.6 (10.0)		3.0 ± 6.1 (2.0)	
Collapse to EMS response (minutes)	11.3 ± 9.7 (9.0)		10.9 ± 9.9 (9.0)		11.7 ± 9.7 (10.0)		11.0 ± 10.8 (8.0)		12.4 ± 11.6 (9.0)	
Collapse to first shock^a ^(minutes)	13.1 ± 6.2 (12.0)		12.8 ± 6.4 (12.0)		13.4 ± 6.0 (12.0)		12.4 ± 6.2 (11.0)		13.1 ± 6.4 (12.0)	
Call to EMS response (minutes)	7.3 ± 4.5 (6.0)		7.0 ± 4.4 (6.0)		7.7 ± 4.5 (7.0)		7.2 ± 5.5 (6.0)		7.2 ± 4.2 (6.0)	

Data presented as *n *or mean ± standard deviation (median). Group A, advanced cardiac life support (ACLS) by emergency life-saving technicians (ELSTs) without bystander-initiated cardiopulmonary resuscitation (BCPR); Group B, ACLS by ELSTs with BCPR; Group C, ACLS by physicians without BCPR; Group D, ACLS by physicians with BCPR. CPR, cardiopulmonary resuscitation; EMS, emergency medical service. ^a^The mean time from collapse to first shock was only calculated in patients who received a shock.

Table 2 presents the 1-month survival rates and CPC rates of all the bystander-witnessed OHCA patients and those with initial VF of cardiac origin. Among the 95,072 bystander-witnessed OHCA patients, the 1-month survival was 8.1%, including 2.9% with good performance and 3.3% with vegetative status or worse. The rate of patients with vegetative status or worse among all survivors was 41.4% (3,171 out of 7,722). Significant differences were found in the 1-month survival and good cerebral performance between Group A and Group B (*P *< 0.01), between Group C and Group D (*P *< 0.01) and between Group B and Group D (*P *< 0.01). In comparisons of Group A and Group C, significant increases were found in the 1-month survival (6.7% vs. 11.6%, *P *< 0.01) and vegetative status or worse (3.1% vs. 6.1%, *P *< 0.01), while the difference in good performance was not significant (1.9% vs. 2.7%, *P *= 0.41). Among the 11,970 bystander-witnessed OHCA patients with initial VF of cardiac origin, the 1-month survival was 24.3%, including 12.0% with good performance and 6.4% with vegetative status or worse. Differences among the groups were similar to all the bystander-witnessed OHCA patients, and the rate of vegetative status or worse was highest in Group C (11.8%).

**Table 2:** One-month outcomes of bystander-witnessed OHCAs and those with initial VF of cardiac origin

	All		Group A		Group B		Group C		Group D	
					
	*n*	%	*n*	%	*n*	%	*n*	%	*n*	%
All bystander-witnessed OHCAs	95,072		53,482		38,077		1,597		1,916	
1-month survival	7,722	8.1	3,608	6.7	3,642	9.6	185	11.6	287	15.0
CPC at 1 month										
Good performance	2,754	2.9	1,026	1.9	1,562	4.1	43	2.7	123	6.4
Moderate disability	659	0.7	317	0.6	294	0.8	17	1.1	31	1.6
Severe cerebral disability	1,117	1.2	560	1.0	489	1.3	28	1.8	40	2.1
Vegetative status or worse	3,171	3.3	1,694	3.2	1289	3.4	97	6.1	91	4.7
Bystander-witnessed OHCAs with initial VF of cardiac origin	11,970		5,840		5,518		229		383	
1-month survival	2,903	24.3	1,247	21.4	1,443	26.2	74	32.3	139	36.3
CPC at 1 month										
Good performance	1,436	12.0	524	9.0	810	14.7	27	11.8	75	19.6
Moderate disability	297	2.5	137	2.3	130	2.4	10	4.4	20	5.2
Severe cerebral disability	395	3.3	197	3.4	173	3.1	10	4.4	15	3.9
Vegetative status or worse	771	6.4	387	6.6	329	6.0	27	11.8	28	7.3

One-month outcomes of all the bystander-witnessed out-of-hospital cardiac arrests (OHCAs) (*n *= 95,072) and those with initial ventricular fibrillation (VF) of cardiac origin (*n *= 11,970). Group A, advanced cardiac life support (ACLS) by emergency life-saving technicians (ELSTs) without bystander-initiated cardiopulmonary resuscitation (BCPR); Group B, ACLS by ELSTs with BCPR; Group C, ACLS by physicians without BCPR; Group D, ACLS by physicians with BCPR. CPC, cerebral performance category.

Table 3 presents the results of logistic regression analyses for the 1-month outcomes of all the bystander-witnessed OHCA patients and those with initial VF of cardiac origin. Among all of the bystander-witnessed OHCA patients, lower rates of overall survival and neurologically good-performance survival were significantly associated with older age and longer call-response interval. The rate of good-performance survival was significantly higher in Group B than in Group A (odds ratio (OR), 2.23; *P *< 0.001), but did not differ significantly between Group A and Group C (OR, 1.18; *P *= 0.317). Group D showed the highest good-performance survival (OR, 2.80; *P *< 0.001). The occurrence of vegetative status or worse was highest in Group C (OR, 1.87; *P *< 0.001). Similar trends were seen in patients with initial VF of cardiac origin, among whom Group D showed significantly improved good-performance survival (OR, 2.06; *P *< 0.001) while Group C showed a significantly higher occurrence of vegetative status or worse (OR, 1.56; *P *= 0.016).

**Table 3:** Logistic regression analyses for 1-month outcomes for bystander-witnessed OHCAs and those with initial cardiac VF

	1-month survival			Good performance at 1 month			Vegetative status or *de facto *brain death at 1 month		
			
	OR	95% CI	*P*	OR	95% CI	*P*	OR	95% CI	*P*
All bystander-witnessed OHCAs									
Age (10-year increase)	0.87	0.86 to 0.89	< 0.001	0.78	0.76 to 0.79	< 0.001	0.96	0.95 to 0.98	< 0.001
Sex									
Male	Ref.			Ref.			Ref.		
Female	1.04	0.99 to 1.10	0.109	0.93	0.84 to 1.01	0.094	1.12	1.04 to 1.21	0.004
Call to EMS response (minutes)	0.90	0.89 to 0.91	< 0.001	0.87	0.86 to 0.89	< 0.001	0.93	0.92 to 0.94	< 0.001
Causes of arrest									
Noncardiac origin	Ref.			Ref.			Ref.		
Cardiac origin with initial non-VF	0.65	0.62 to 0.69	< 0.001	1.12	1.00 to 1.25	0.050	0.54	0.49 to 0.59	< 0.001
Cardiac origin with initial VF	3.57	3.37 to 3.79	< 0.001	6.41	5.82 to 7.07	< 0.001	1.66	1.51 to 1.82	< 0.001
Combination of BCPR and ACLS									
ACLS by ELSTs without BCPR	Ref.			Ref.			Ref.		
ACLS by ELSTs with BCPR	1.51	1.43 to 1.59	< 0.001	2.23	2.05 to 2.42	< 0.001	1.09	1.02 to 1.18	0.018
ACLS by physicians without BCPR	1.63	1.39 to 1.92	< 0.001	1.18	0.86 to 1.61	0.317	1.87	1.52 to 2.32	< 0.001
ACLS by physicians with BCPR	2.17	1.89 to 2.49	< 0.001	2.80	2.28 to 3.43	< 0.001	1.45	1.17 to 1.80	0.001
Bystander-witnessed OHCAs with initial VF of cardiac origin									
Age (10-year increase)	0.85	0.83 to 0.86	< 0.001	0.79	0.77 to 0.81	< 0.001	0.99	0.95 to 1.03	0.669
Sex									
Male	Ref.			Ref.			Ref.		
Female	1.17	1.07 to 1.27	< 0.001	1.09	0.97 to 1.22	0.137	1.14	0.99 to 1.32	0.076
Call to EMS response (minutes)	0.90	0.89 to 0.91	< 0.001	0.90	0.88 to 0.92	< 0.001	0.93	0.91 to 0.95	< 0.001
Combination of BCPR and ACLS									
ACLS by ELSTs without BCPR	Ref.			Ref.			Ref.		
ACLS by ELSTs with BCPR	1.18	1.09 to 1.26	< 0.001	1.30	1.18 to 1.43	< 0.001	1.00	0.88 to 1.14	0.993
ACLS by physicians without BCPR	1.87	1.49 to 2.34	< 0.001	1.60	1.20 to 2.14	0.002	1.56	1.09 to 2.24	0.016
ACLS by physicians with BCPR	2.10	1.72 to 2.56	< 0.001	2.06	1.62 to 2.63	< 0.001	1.28	0.90 to 1.83	0.167

Logistic regression analyses for 1-month outcomes for all the bystander-witnessed out-of-hospital cardiac arrests (OHCAs) (*n *= 95,072) and those with initial ventricular fibrillation (VF) of cardiac origin (*n *= 11,970). ACLS, advanced cardiac life support; BCPR, bystander-initiated cardiopulmonary resuscitation; CI, confidence interval; ELST, emergency life-saving technician; EMS, emergency medical service; OR, odds ratio; Ref., reference.

Table 4 presents the demographics and outcomes of patients whose arrests were witnessed by EMS providers, and Table 5 presents logistic regression analyses for these patients. The group treated with ACLS by physicians showed higher overall survival (OR, 1.27; *P *= 0.013) and good-performance survival (OR, 1.47; *P *< 0.001), while the occurrence of vegetative status or worse did not differ between the two groups (OR, 0.92; *P *= 0.646).

**Table 4:** Baseline characteristics and 1-month outcomes of patients whose arrests witnessed by EMS providers

	All (*n *= 23,127)		ACLS by ELSTs (*n *= 22,131)		ACLS by physicians (*n *= 996)	
			
	*n*	%	*n*	%	*n*	%
Sex (males)	14,434	62.4	13,797	62.3	637	64.0
Age (years)	69.6 ± 18.0		69.8 ± 17.9		65.2 ± 20.0	
Causes of arrest						
Cardiac origin	12,024	52.0	11,503	52.0	521	52.3
Noncardiac origin	11,103	48.0	10,628	48.0	475	47.7
Initial rhythm						
Ventricular fibrillation	1,918	8.3	1,818	8.2	100	10.0
Pulseless electrical activity	8,424	36.4	8,092	36.6	332	33.3
Asystole	4,471	19.3	4,325	19.5	146	14.7
Others	8,314	35.9	7,896	35.7	418	42.0
Call to EMS response (minutes)	6.9 ± 4.3		6.9 ± 4.2		6.9 ± 5.3	
Outcomes						
1-month survival	2,642	11.4	2,493	11.3	149	15.0
CPC at 1 month						
Good performance	1,325	5.7	1,235	5.6	90	9.0
Moderate disability	234	1.0	223	1.0	11	1.1
Severe cerebral disability	312	1.3	295	1.3	17	1.7
Vegetative status or worse	765	3.3	734	3.3	31	3.1

Data presented as *n *or mean ± standard deviation. ACLS, advanced cardiac life support; CPC, cerebral performance category; ELST, emergency life-saving technician; EMS, emergency medical service.

**Table 5:** Logistic regression analyses for 1-month outcomes for OHCAs witnessed by EMS providers (***n *= **23,127)

	1-month survival			Good performance at 1 month			Vegetative status or *de facto *brain death at 1 month		
			
	OR	95% CI	*P*	OR	95% CI	*P*	OR	95% CI	*P*
Age (10-year increase)	0.89	0.87 to 0.91	< 0.001	0.80	0.78 to 0.83	< 0.001	0.97	0.93 to 1.01	0.119
Sex									
Male	Ref.			Ref.			Ref.		
Female	1.02	0.94 to 1.12	0.617	0.90	0.79 to 1.02	0.097	1.18	1.02 to 1.37	0.028
Causes of arrest									
Noncardiac origin	Ref.			Ref.			Ref.		
Cardiac origin with initial non-VF	1.58	1.43 to 1.73	< 0.001	3.00	2.58 to 3.49	< 0.001	0.93	0.80 to 1.09	0.360
Cardiac origin with initial VF	7.58	6.68 to 8.59	< 0.001	17.82	15.07 to 21.07	< 0.001	1.36	1.04 to 1.77	0.024
Advanced cardiac life support									
By ELSTs	Ref.			Ref.			Ref.		
By physicians	1.27	1.05 to 1.53	0.013	1.47	1.16 to 1.87	0.002	0.92	0.64 to 1.32	0.646

CI, confidence interval; ELST, emergency life-saving technician; EMS, emergency medical service; OHCA, out-of-hospital cardiac arrest; OR, odds ratio; Ref., reference; VF, ventricular fibrillation.

## Discussion

Survival from OHCA remains poor in most countries [2-8]. Numerous studies have suggested that BCPR is the most fundamental factor for improving the survival of OHCA patients [3-6,9]. In the present study, BCPR significantly improved survival with good cerebral performance, and an independent effect of BCPR for OHCA patients was demonstrated.

One of the significant findings of the present study was the improvement of both the overall 1-month survival and good neurological performance at 1 month in patients with ACLS by physicians. A previous report showed that a physician on board an ACLS unit was not an independent factor for improved survival, but the study only included 539 OHCA patients in a limited area [13]. To the best of our knowledge, the present study is the first to demonstrate the survival-improving effects of physician-manned ACLS in a nationwide setting.

Physician-staffed ambulances are in use in many European nations [9,13,21,22], while paramedics in the United States are permitted to provide highly advanced support partially because physician-manned ambulances are considered an inefficient use of physician resources [9]. In Japan, extending the physician-staffed ambulance system may be practically difficult because of the shortage of emergency physicians [17].

Another important finding of the present study was the confirmation of high survival with poor neurological outcomes of bystander-witnessed OHCA patients treated with ACLS by physicians without BCPR. ACLS by physicians can reinforce the effects of preceding BCPR. In other words, the combination of BCPR and ACLS by physicians is considered the best way to achieve a patient's comeback from collapse and subsequent well-being. In the cases without BCPR, however, the crucial delay in receiving first aid presumably caused many survivors to suffer irreparable brain damage. Life support by physicians without preceding BCPR may cause an increase in unfavorable, and sometimes desperate, consequences. Our eyes must be opened to the fact that more than 40% of 1-month survivors among the bystander-witnessed OHCA patients were classified as vegetative status or *de facto *brain death. Great care should be taken of the fact that more than 50% of 1-month survivors among the bystander-witnessed OHCA patients who underwent ACLS by physicians without BCPR were of vegetative status or *de facto *brain death. These are the realities of EMS procedures for OHCA patients.

Of course, it is not the physician intervention that is detrimental, but the duration of no blood flow. Our study clearly shows that priority should be given to the enhancement of BCPR to improve the neurologically favorable outcomes of OHCA patients. Teaching on how to behave in emergency situations is a common public health problem worldwide. Training programs in the school curriculum could be the best way to train the whole population [23,24]. In Japan, CPR training is performed for millions of citizens each year, and new driver license applicants have recently been obliged to undergo CPR training programs at driving schools [14]. Nevertheless, our study revealed that BCPR only occurred in 42% of bystander-witnessed OHCA cases in Japan. While this figure is relatively high compared with many countries [2,25], there is much room for improvement. Efforts should be made to improve the quantity and quality of BCPR. Ideally, ACLS by physicians should be linked with preceding BCPR.

Several limitations of the present study should be acknowledged. A randomized trial is not feasible for this type of study owing to ethical and informed consent issues. Furthermore, a large-scale randomized trial looking at physician EMS versus nonphysician EMS is very challenging to perform. Although the groups were large, the present study was based on a nonrandomized observational study and thus jeopardized by several potential biases. First, and most importantly, prehospital physician EMS was only available in limited geographical areas around specific emergency medical centers. Although our data lacked information on the hospitals receiving the patients, it appears likely that most patients in the physician EMS groups (Group C and Group D) were brought to the specific hospitals to which the physicians on board were affiliated. It also seems likely that a relationship between physician EMS and outcomes could partly reflect differences in post-return of spontaneous circulation treatments available at receiving hospitals; including therapeutic hypothermia [26-28], percutaneous coronary intervention, and a focus on goal-directed treatment for the reperfusion period [28]. These treatments are only available at some centers, potentially influencing outcomes. We were unable to adjust for this factor because we had no information on what treatments were performed at the receiving hospitals. Second, similar to all registry-based surveys, the validity and integrity of the data were potential limitations, although they were minimized by the large sample size collected with the population-based design. Finally, long-term outcomes such as the rate of discharge from hospital or 1-year survival could not be assessed.

## Conclusions

BCPR significantly improved the survival of OHCA patients with good cerebral performance. The combination of BCPR and ACLS by physicians was the best way to improve the outcomes. ACLS by physicians without preceding BCPR, however, increased the incidence of neurologically unfavorable outcomes. Priority should be given to the enhancement of BCPR, and ACLS by physicians should ideally be linked with preceding BCPR.

## Key messages

• Among 95,072 patients with bystander-witnessed OHCA, 7,722 (8.1%) patients were alive at 1 month, including 2,754 (2.9%) with good performance and 3,171 (3.3%) with vegetative status or worse.

• More than 40% of 1-month survivors were classified as vegetative status or *de facto *brain death.

• The combination of BCPR and ACLS by physicians was the best way to improve outcomes.

• Life support by physicians without preceding BCPR increased the occurrence of vegetative status or worse.

## Abbreviations

ACLS: advanced cardiac life support; BCPR: bystander-initiated cardiopulmonary resuscitation; CPC: cerebral performance category; CPR: cardiopulmonary resuscitation; ELST: emergency life-saving technician; EMS: emergency medical service; FDMA: Fire and Disaster Management Agency of Japan; OHCA: out-of-hospital cardiac arrest; OR: odds ratio; VF: ventricular fibrillation.

## Competing interests

The authors declare that they have no competing interests.

## Authors' contributions

HY, HH, ST, MA, TO, SK and TI participated in the idea formation, study design, data analyses, interpretation of results and writing of the report. All the authors read and approved the final manuscript.
